# Cancer survival in parents who lost a child: a nationwide study in Denmark

**DOI:** 10.1038/sj.bjc.6600948

**Published:** 2003-05-27

**Authors:** J Li, C Johansen, J Olsen

**Affiliations:** 1 The Danish Epidemiology Science Centre, Department of Epidemiology and Social Medicine, University of Aarhus, Vennelyst Boulevard 6, DK-8000 Aarhus C, Denmark; 2 Department of Psychosocial Cancer Research, Institute of Cancer Epidemiology, Danish Cancer Society, Strandboulevarden 49, DK-2100 Copenhagen, Denmark

**Keywords:** survival, psychological stress, bereavement, epidemiology

## Abstract

Psychological stress has been suggested to shorten cancer survival, but few studies have examined the effect of parental bereavement, and the results have been inconsistent. We identified all 21 062 parents who lost a child in Denmark from 1980 to 1996 and among them, 1630 parents with subsequent incident cancer formed the exposed cohort. We recruited 6237 incident cancer patients from a group of 293 745 randomly selected unexposed parents matched on family structure at the same time as the bereaved parents. All incident cancers in the two cohorts were followed to the end of 1997, or until they died. Cox proportional-hazards regression models were used to evaluate the hazard ratio (HR) of dying in exposed parents with cancer. The overall HR of dying from an incident cancer in exposed parents was 1.23 (95% confidence interval 1.03–1.47) compared to parents with cancer who did not lose a child. The HRs were nearly identical to those in the unexposed parents for site-specific cancers like lung cancer, breast cancer, and other groups of cancers like cancers in all digestive organs, smoking-related cancers, alcohol-related cancers, hormone-related cancers, virus/immune-related cancers, and lymphatic/haematopoietic cancers. Death of a child is not a strong prognostic factor for cancer survival among parents diagnosed with cancer after the bereavement. However, a small impairment in overall cancer survival cannot be ruled out.

Psychological stress may alter immune function that could influence tumour growth and metastasis (Cohen and Rabin, 1998; Kiecolt-Glaser and Glaser, 1999), but whether stress is associated with cancer survival is not clear (Funch and Marshall, 1983; Jamison *et al*, 1987; Levy *et al*, 1988; Tross *et al*, 1996; Levav *et al*, 2000). It has been proposed recently that stress may have no obvious effect on overall cancer survival, but could affect survival for specific cancers (Andersen *et al*, 1994; Kvikstad *et al*, 1995). However, previous studies have often been flawed by inadequate control of confounders, loss to follow-up, small sample sizes, and lack of tumour-specific information (Ross *et al*, 2002). Furthermore, self-reported stress in most previous studies is vulnerable to bias (Macleod *et al*, 2002).

The death of a child is one of the greatest stresses (American Psychiatric Association, 1987), and may produce depression, despair, anxiety, guilt, anger, hostility, and hopelessness (Osterweis *et al*, 1984; Rubin and Malkinson, 2001). In addition, bereaved parents may have more somatic complaints, interpersonal difficulties, and react with more adverse health behaviours (Osterweis *et al*, 1984; Anda *et al*, 1990; Camacho *et al*, 1991; Rubin and Malkinson, 2001), which could be detrimental to health. Few studies have explored the health consequences of parental bereavement on cancer survival (Kvikstad and Vatten, 1996; Levav *et al*, 2000).

We investigated the effect of the death of a child on the overall and specific cancer survival in parents who lost a child. By using the data from nationwide registers, we had the opportunities to collect accurate information on exposure and follow-up, and detailed data on socioeconomic factors for confounder control.

## MATERIALS AND METHODS

### Study design and data collection

The cohort has been described elsewhere (Li *et al*, 2002). In short, we recruited all 21 062 parents who lost a child from 1980 to 1996, together with 293 745 randomly selected parents who had not lost a child. Among the bereaved parents, 461 had a subsequent incident cancer and they were selected to the exposed cohort. Among the parents who did not lose a child, 6237 had an incident cancer after the recruitment and they were included into the unexposed cohort. We collected information on cohort members (date of birth, date of death, gender, school education, residence, cause of death, cancer incidence) from the Prevention Register and The Danish Cancer Registry (Roed *et al*, 1999; Danish National Board of Health, 2000). Identification and linkage was based upon the unique personal identification number assigned to all residents in Denmark since 1968 (Roed *et al*, 1999).

For cancer survival, follow-up started when participants were diagnosed with cancer and ended on date of death, emigration, or 31 December 1997, whichever came first. The main outcomes of interest were overall cancer survival, survival for site-specific cancers, and five subgroups of cancers in which stress could play a role in the aetiology: smoking-related cancers (ICD7 codes 140, 141, 143–150, 157, 160–162, 180, and 181), alcohol-related cancers (ICD7 codes 141, 143–146, 148–150, 155, and 161), virus and immune-related cancers (ICD 7 codes 155, 171, 191, 200–202, 204), lymphatic/haematopoietic tissue cancers (ICD 7 codes 200–205) and hormone-related cancers (ICD 7 codes 170, 172, 175, 177).

### Statistical analysis

Cox proportional-hazards regressions were fitted to obtain the hazard ratios of dying with cancer, and to estimate 95% confidence intervals (CIs). Sociodemographic characteristics of the cohort members, such as age at entry (<30, 30–39, 40+years), sex (male, female), school education (basic, secondary or higher, no information), and residence (cities with a population of more than 100 000 inhabitants, other places) were included in the models as potential confounders.

We examined the relative risk in cancer survival between exposed and unexposed cohort members by length of time from the study entry (exposure) to cancer diagnosis (0–4, 5–8, 9–12, ⩾12 years), and also according to age, gender, and type of death of the deceased child (unexpected death (sudden death by unknown cause, ICD8 codes 795.0–795.9, ICD 10 codes R95–R97, motor vehicle accidents, ICD8 codes 810.0–823.0, ICD10 codes V01–V89, suicide, ICD8 codes 950.0–959.9, ICD10 codes X60–X84, other accidents and violence, ICD8 codes 800.0–807.9, 825.0–949.9, 960.0–999.9, ICD10 codes V90–V99, W00–X59, X85-Y89) or death by other causes).

Similar analytic strategies were also applied to subgroups of cancers, and specific cancers with short or long expected survival (International Agency for Research on Cancer, World Health Organization, European Commision, 1999).

## RESULTS

A total of 135 parents died of cancer among 461 incident cases in the exposed cohort, and 1630 parents died of cancer among 6237 incident cases in the unexposed group. The overall hazard ratios (HRs) for dying from cancer for both parents, fathers, and mothers in the exposed cohort were 1.23 (95% confidence interval 1.03–1.47), 1.26 (0.98–1.63), and 1.19 (0.93–1.52), respectively. We found no clear indication of a higher relative mortality rate in cancer patients diagnosed shortly after the bereavement (Table 1

**Table 1:** Hazard ratio (HRs) of dying in bereaved cancer parents grouped by the time from recruitment to cancer diagnosis, Denmark 1980-1997

	**HR (95%CI)**		
**Time from onset of bereavement to cancer diagnosis**	**Parents**	**Fathers**	**Mothers**
All	1.23 (1.03–1.47)	1.26 (0.98–1.63)	1.19 (0.93–1.52)
0–4 years	1.15 (0.79–1.69)	1.59 (0.89–2.85)	0.93 (0.56–1.57)
5–8 years	1.28 (0.92–1.77)	1.32 (0.82–2.12)	1.26 (0.81–1.99)
9–12 years	1.31 (0.93–1.85)	1.16 (0.67–2.01)	1.45 (0.93–2.25)
>12 years	1.15 (0.78–1.69)	1.21 (0.76–1.95)	1.11 (0.58–2.13)

a HR=hazard ratio adjusted for: age group, gender, school education, residence, number of children in the family, number of parents in the family

).

Age, gender, and cause of death of the deceased child did not significantly modify cancer survival between exposed and unexposed parents (data not shown). The HR of dying from cancer did not differ in bereaved parents according to age, school education, residence place, the number of children in the family, or the number of parents in the family (data not shown).

Table 2

**Table 2:** Hazard ratios (HRs) of dying in cancer parents by cancer groups, Denmark 1980–1997

	**HR (95% CI)**		
**Cancer groups**	**Parents**	**Fathers**	**Mothers**
Cancer in the digestive system	1.23 (0.85–1.78)	0.97 (0.60–1.56)	1.22 (0.70–2.13)
Cancers in the respiratory system	0.95 (0.65–1.40)	0.84 (0.52–1.34)	0.92 (0.47–1.77)
Smoking-related cancers	1.02 (0.74–1.40)	0.99 (0.66–1.48)	1.06 (0.66–1.76)
Alcohol-related cancers	1.74 (0.80–3.77)	1.99 (0.81–4.85)	1.59 (0.30–8.41)
Virus/immune-related cancers	1.08 (0.67–1.73)	0.96 (0.46–1.98)	0.99 (0.55–1.79)
Lymphatic/haematopoietic cancers	0.86 (0.39–1.88)	1.20 (0.47–3.09)	0.45 (0.11–1.96)
Hormone-related cancers (mothers)			1.36 (0.91–2.04)

a HR=hazard ratio adjusted for: age group, gender, school education, residence, number of children in the family, number of parents in the family

b Smoking-related cancers=ICD 7 codes 140, 141, 143–149, 150, 157, 160–162, 180, 181; alcohol-related cancers=ICD 7 codes 141, 143–146, 148-150, 155, 161; virus/immune-related cancers=ICD 7 codes 155, 171, 191, 200–202, 204; lymphatic/haematopoietic cancers=ICD 7 codes 200–205; hormone-related cancers=ICD 7 codes 170, 172, 175, 177.

shows survival for cancers in digestive organs, respiratory organs, and selected cancer groups. We did not observe any statistically significant increased HRs in any of these groups. Similar results were seen for site-specific cancers classified according to their expected survival (Table 3

**Table 3:** Hazard ratios (HRs) of dying in parents for specific cancers grouped according to, *a priori*, expected 5-year survival^b^: Cox regression

**Cancer sites and expected 5-year survival**	**Cases in exposed/unexposed**	**Deaths in exposed/unexposed**	**HR (95% CI)**
*Cancers with <40% 5-year survival*	81/849	54/596	0.96 (0.73–1.28)
Oesophagus	5/36	5/22	1.22 (0.33–4.51)
Gall bladder	3/22	3/12	1.54 (0.18–12.93)
Stomach	10/101	8/65	1.42 (0.65–3.09)
Lung cancer	46/487	29/354	0.85 (0.57–1.25)
Acute leukaemia	9/69	3/41	0.59 (0.13–2.63)
Pancreas	8/100	6/79	1.92 (0.75–4.89)
			
*Cancers with 40–75% 5-year survival*	183/2519	45/548	1.29 (0.93–1.72)
Breast (mothers)	99/1380	20/244	1.40 (0.89–2.22)
Bone and soft tissue	5/58	1/12	0.33 (0.02–6.24)
Colon/rectum	21/253	6/90	0.89 (0.38–2.08)
Ovary (mothers)	12/173	6/67	1.76 (0.72–4.29)
Cervix (mothers)	36/477	7/63	1.33 (0.60–2.95)
Prostate (fathers)	5/111	2/57	4.99 (0.72–34.76)
			
*Other cancers not included in the above two groups*	221/3122	36/486	1.19 (0.84–1.67)

a HR: hazard ratio adjusted for: age group, gender, school education, residence, number of children in the family, number of parents in the family.

b Expected 5-year survival: specific cancers grouped according to the grouping in The EUROCARE-2 Study, 1985–89 for aged 15–44 years as following: <40% (hypopharynx, oesophagus, liver, gall bladder, pancreas, lung, pleura, stomach, acute leukaemia); 40–75% (colon/rectum, cervix, bone and soft tissue, ovary, prostate, larynx, breast, brain, kidney); other cancers not included in the above two groups.

).

## DISCUSSION

We observed that parents who lost a child had a slightly shorter cancer survival than those who did not lose a child. This slightly shorter survival was not observed in any particular cancer group, nor was it associated with the sex or age of the parents, the type of bereavement, or the time since exposure. The effect could be causal or because of uncontrolled confounding.

The possible link between psychological stress and cancer survival has generated a literature of contradictory findings. Some earlier studies suggested worse survival for breast cancer patients exposed to stress (Funch and Marshall, 1983; Levy *et al*, 1988). However, these associations have been weak because of methodological limitations, such as insufficient power and inadequate assessment of exposure were present in those studies. Other studies with more tightly defined patient populations, more adequate control for medical and treatment-related factors, failed to provide evidence for a positive association (Jamison *et al*, 1987; Tross *et al*, 1996). Another study including 14 669 cancer patients in bereaved middle-aged mothers showed no reduced survival for overall and site-specific cancer survival. The results were similar when stratifying on metastatic status (Kvikstad and Vatten, 1996). In one recent study, cancer survival was not affected by the death of a child among 677 bereaved parents (Levav *et al*, 2000). The results of our study are in line with these negative findings, suggesting that psychological stress does not play any major role in cancer survival.

We have previously shown that the death of a child was associated with a slightly increased cancer risk in mothers and suggested that stress-related health behaviours may account for this increased risk (Li *et al*, 2002). The observed weak associations in this and the previous study (Li *et al*, 2002) may be because of adaptive lifestyle factors related to stress exposure, or poorer compliance to the medical treatment of the cancer diseases. In addition, disease-related variables or treatment effect, to which we did not have access, may outweigh the influence of psychological stress, if any, on survival (Jamison *et al*, 1987; Buddeberg *et al*, 1991; Tross *et al*, 1996).

Parents' response to the death of a child could vary with the elapsed time, type of bereavement, and personal characteristics (Osterweis *et al*, 1984; Rubin and Malkinson, 2001). We found, however, no trend in the risk of dying according to time since bereavement, which is congruent with one other study (Levav *et al*, 2000). We did not observe any effect of age and gender of the deceased child, or effect of sudden unexpected bereavement, which may be related to the health outcomes in bereaved parents (Osterweis *et al*, 1984; Parkes, 1998). Personal characteristics such as age, gender, and education were not associated with modified HRs of dying in the cancer patients, either.

Psychological stress has been suggested to play a role in cancer progression through immune downregulation, poorer repair of damaged DNA, and alterations in apoptosis (Cohen and Rabin, 1998; Kiecolt-Glaser and Glaser, 1999). Some have suggested that stress is involved in the causation of breast, colo-rectum, haematopoietic and lymphatic, immune-related, and hormone-related malignancies (Gallo *et al*, 2000; Levav *et al*, 2000). We observed no reduced survival for those cancers in the bereaved parents. Bereaved persons are often subjected to more adverse health behaviours (Anda *et al*, 1990; Camacho *et al*, 1991). However, no reduced survival was found for the digestive system or respiratory cancers. Furthermore, we did not observe any difference in relative mortality rates for cancers with different, *a priori*, expected survival time.

Our study has a number of advantages. We had access to valid classifications of exposure, and complete follow-up (Danish National Board of Health, 2000; Knudsen, 1998; Roed *et al*, 1999). We used the death of a child as the stress indicator, which has been described as the most intense of all grief with a long-lasting stress effect (Osterweis *et al*, 1984; Rubin and Malkinson, 2001). All bereaved parents in Denmark during the study period were enrolled into the exposed cohort. In addition, all data used in our study were extracted from the databases that were collected independently of the research hypothesis, which minimises the risk of surveillance bias that often is a problem in follow-up studies. Furthermore, we were able to study both overall cancer survival and survival for specific cancers.

Our study also has limitations. Firstly, we had no information on the stage of the disease when they were diagnosis or for cancer treatment, which are the most important factors for cancer progression. Secondly, we had no information on health behaviours after cancer diagnosis, or compliance to the treatment. Thirdly, the enrolled parents were young and had a low risk of cancer, which yielded limited statistical power especially for subgroups of cancer.

In summary, we found a slightly reduced survival in bereaved parents and this may be due to confounding. The effect of psychological stress related to bereavement on cancer survival is likely to be small, if any effect exists at all.

## References

[bib1] American Psychiatric Association (1987) Diagnostic and Statistical Manual of Mental Disorders. Washington, DC: National Academy Press

[bib2] Anda RF, Williamson DF, Escobedo LG, Mast EE, Giovino GA, Remington PL (1990) Depression and the dynamics of smoking. A national perspective. JAMA. 264: 1541–1545 2395193

[bib4] Andersen BL, Kiecolt-Glaser JK, Glaser R (1994) A biobehavioral model of cancer stress and disease course. Am Psychol 49: 389–404 8024167 10.1037//0003-066x.49.5.389 PMC2719972

[bib5] Buddeberg C, Wolf C, Sieber M, Riehl-Emde A, Bergant A, Steiner R, Landolt-Ritter C, Richter D (1991) Coping strategies and course of disease of breast cancer patients Results of a 3-year longitudinal study. Psychother Psychosom 55: 151–157 1891562 10.1159/000288423

[bib6] Camacho TC, Roberts RE, Lazarus NB, Kaplan GA, Cohen RD (1991) Physical activity and depression: evidence from the Alameda County Study. Am J Epidemiol 134: 220–231 1862805 10.1093/oxfordjournals.aje.a116074

[bib7] Cohen S, Rabin BS (1998) Psychologic stress, immunity, and cancer. J Natl Cancer Inst 90: 3–4 9428772 10.1093/jnci/90.1.3

[bib8] Danish National Board of Health (2000) Cancer Incidence in Denmark 1997. Copenhagen Danish National Board of Health

[bib9] Funch DP, Marshall J (1983) The role of stress, social support and age in survival from breast cancer. J Psychosom Res 27: 77–83 6834302 10.1016/0022-3999(83)90112-5

[bib10] Gallo JJ, Armenian HK, Ford DE, Eaton WW, Khachaturian AS (2000) Major depression and cancer: the 13-year follow-up of the Baltimore epidemiologic catchment area sample (United States). Cancer Causes Control 11: 751–758 11065012 10.1023/a:1008987409499

[bib12] International Agency for Research on Cancer World Health Organization, European Commission (1999) Survival of Cancer Patients in Europe: the EUROCARE-2 Study. Lyon: Oxford University Press

[bib13] Jamison RN, Burish TG, Wallston KA (1987) Psychogenic factors in predicting survival of breast cancer patients. J Clin Oncol 5: 768–772 3572466 10.1200/JCO.1987.5.5.768

[bib14] Kiecolt-Glaser JK, Glaser R (1999) Psychoneuroimmunology and cancer: fact or fiction? Eur J Cancer 35: 1603–1607 10673969 10.1016/s0959-8049(99)00197-5

[bib15] Knudsen LB (1998) The Danish Fertility Database. Dan Med Bull 45: 221–225 9587707

[bib16] Kvikstad A, Vatten LJ (1996) Risk and prognosis of cancer in middle-aged women who have experienced the death of a child. Int J Cancer 67: 165–169 8760581 10.1002/(SICI)1097-0215(19960717)67:2<165::AID-IJC2>3.0.CO;2-R

[bib17] Kvikstad A, Vatten LJ, Tretli S (1995) Widowhood and divorce in relation to overall survival among middle-aged Norwegian women with cancer. Br J Cancer 71: 1343–1347 7779736 10.1038/bjc.1995.261 PMC2033850

[bib18] Levav I, Kohn R, Iscovich J, Abramson JH, Tsai WY, Vigdorovich D (2000) Cancer incidence and survival following bereavement. Am J Public Health 90: 1601–1607 11029995 10.2105/ajph.90.10.1601 PMC1446385

[bib19] Levy SM, Lee J, Bagley C, Lippman M (1988) Survival hazards analysis in first recurrent breast cancer patients: seven-year follow-up. Psychosom Med 50: 520–528 3186895 10.1097/00006842-198809000-00008

[bib20] Li J, Johansen C, Hansen D, Olsen J (2002) Cancer incidence in parents who lost a child. Cancer 95: 2237–2242 12412179 10.1002/cncr.10943

[bib21] Macleod J, Davey SG, Heslop P, Metcalfe C, Carroll D, Hart C (2002) Limitations of adjustment for reporting tendency in observational studies of stress and self reported coronary heart disease. J Epidemiol Community Health 56: 76–77 11801624 10.1136/jech.56.1.76 PMC1732012

[bib22] Osterweis M, Solomon F, Green M (1984) Bereavement: Reactions, Consequences, and Care. Washington, DC: National Academy Press 25032459

[bib23] Parkes CM (1998) Bereavement in adult life. BMJ 316: 856–859 9549464 10.1136/bmj.316.7134.856 PMC1112778

[bib24] Roed AS, Juhl C, Kamper-Jorgensen F (1999) The Danish Prevention Register. A comprehensive health and socio-economic, individual based register. Dan Med Bull 46: 269–272 10421986

[bib25] Ross L, Boesen EH, Dalton SO, Johansen C (2002) Mind and cancer: does psychosocial intervention improve survival and psychological well-being? Eur J Cancer 38: 1447–1457 12110489 10.1016/s0959-8049(02)00126-0

[bib26] Rubin SS, Malkinson R (2001) Parental response to child loss across the life cycle: clinical and research perspecitves. In Handbook of Bereavement Research: Conequences, Coping, and Care Stroebe MS, Hansson RO, Stroebe W (eds) pp 219–239. Washington, DC: American Psychological Association

[bib27] Tross S, Herndon J, Korzun A, Kornblith AB, Cella DF, Holland JF, Raich P, Johnson A, Kiang DT, Perloff M, Norton L, Wood W, Holland JC (1996) Psychological symptoms and disease-free and overall survival in women with stage II breast cancer. Cancer and Leukemia Group B. J Natl Cancer Inst 88: 661–667 8627642 10.1093/jnci/88.10.661

